# Supplementary figures and images for: Transcriptome Profiling of Potato (Solanum tuberosum L.) Responses to Root-Knot Nematode (Meloidogyne javanica) Infestation during A Compatible Interaction
Source: Microorganisms. 2020 Sep 21;8(9):1443. doi: 10.3390/microorganisms8091443 (PMC7563278; doi:10.3390/microorganisms8091443)

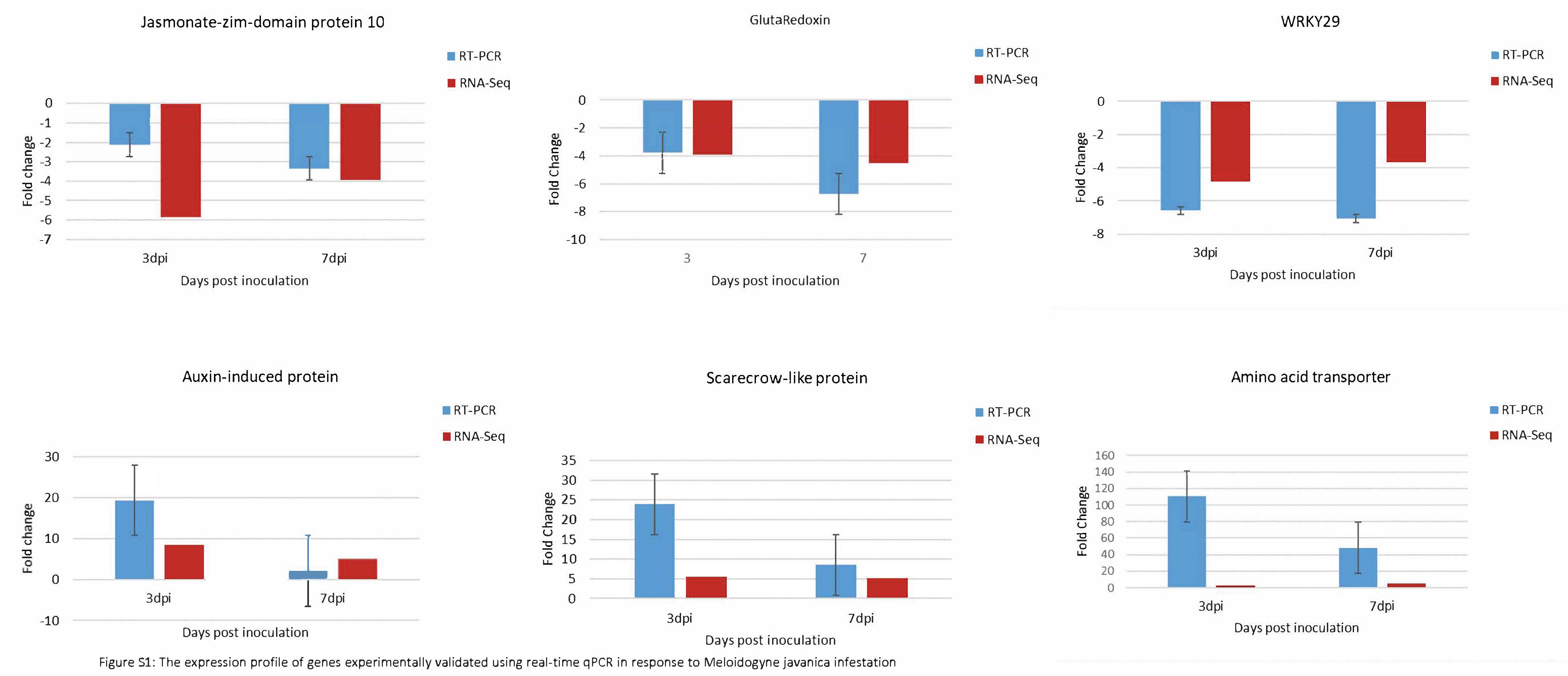

Supplement: Supplementary file 1 [file microorganisms-08-01443-s001.zip › microorganisms-919124-SI/Supplememtary Materials/Figure S1 png.jpg]

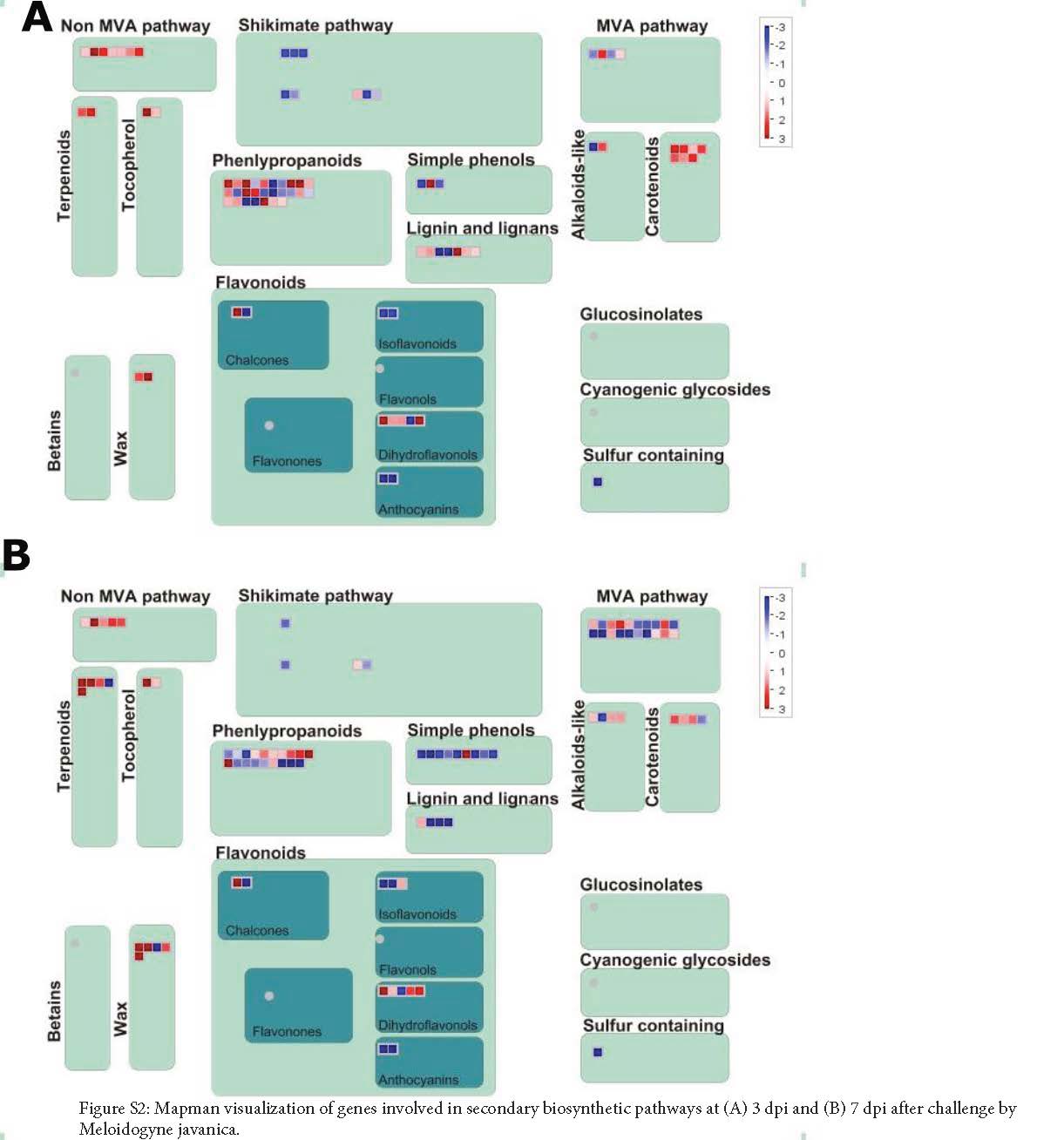

Supplement: Supplementary file 1 [file microorganisms-08-01443-s001.zip › microorganisms-919124-SI/Supplememtary Materials/Figure S2 png.jpg]
